# Diversity of aphantasia revealed by multiple assessments of visual imagery, multisensory imagery, and cognitive style

**DOI:** 10.3389/fpsyg.2023.1174873

**Published:** 2023-07-18

**Authors:** Junichi Takahashi, Godai Saito, Kazufumi Omura, Daichi Yasunaga, Shinichiro Sugimura, Shuichi Sakamoto, Tomoyasu Horikawa, Jiro Gyoba

**Affiliations:** ^1^Faculty of Human Development and Culture, Fukushima University, Fukushima, Japan; ^2^Department of Psychology, Graduate School of Arts and Letters, Tohoku University, Sendai, Japan; ^3^Faculty of Education, Art and Science, Yamagata University, Yamagata, Japan; ^4^Faculty of Letters, College of Human and Social Sciences, Kanazawa University, Kanazawa, Japan; ^5^Department of Psychology, Hiroshima University, Hiroshima, Japan; ^6^Research Institute of Electrical Communication, Tohoku University, Sendai, Japan; ^7^NTT Communication Science Laboratories, Nippon Telegraph and Telephone Corporation, Atsugi, Japan; ^8^Department of Psychology, College of Psychology and Education, Shokei Gakuin University, Natori, Japan

**Keywords:** aphantasia, mental imagery, visual imagery, multisensory imagery, cognitive style, face recognition ability

## Abstract

Aphantasia—a condition wherein individuals have a reduced or absent construction of voluntary visual imagery—is diagnosed using either the Vividness of Visual Imagery Questionnaire (VVIQ) or self-identification. However, a significant discrepancy exists between the proportions of aphantasia in the populations assessed using these two criteria. It is unclear why the reported proportions differ excessively and what percentage of people cannot form visual imagery. We investigated the replicability of the proportion of people with aphantasia using both criteria in the same population of participants. Therefore, we explored the potential causes of the discrepancy and characteristics of putative aphantasia in terms of multisensory imagery, cognitive style, and face recognition ability. First, we conducted an online sampling study (Study 1: *N* = 2,871) using the VVIQ, self-identification of a reduction in visual imagery, Questionnaire upon Mental Imagery (QMI), and Verbalizer-Visualizer Questionnaire (VVQ). We found that 3.7 and 12.1% fulfilled the VVIQ and self-identification criteria, respectively, roughly replicating the proportions reported in previous studies. The self-identification criterion—but not the VVIQ criterion—contains items related to face recognition; hence, we suspected that face recognition ability was factor contributing to this discrepancy and conducted another online sampling study (Study 2: *N* = 774). We found a significant correlation between VVIQ and face recognition ability in the control group with self-identification, but not in the group defined by low VVIQ (VVIQ ≤32). As the participants in the control group with self-identification tended to exhibit moderately high VVIQ scores but low face recognition ability, we reason that the discrepancy can be partially explained by the contamination of individual differences in face recognition ability. Additional analyses of Study 1 revealed that the aphantasia group included participants who lacked all types of sensory imagery or only visual imagery in multisensory imagery and exhibited a non-specific cognitive style. This study indicates that the VVIQ alone may be insufficient to diagnose individuals who report an inability to form visual imagery. Furthermore, we highlight the importance of multiple assessments—along with the VVIQ—to better understand the diversity of imagery in aphantasia.

## 1. Introduction

Visual imagery refers to a set of representations that create the experience of viewing a stimulus in the absence of appropriate sensory input (Kosslyn, 2005). Furthermore, visual imagery is widely used in daily life through processes such as perception, memory, and thinking. Recently, a mental condition wherein individuals exhibit a reduction in—or absence of—visual imagery has been reported. This condition is termed aphantasia (Zeman et al., 2015).

Indeed, various definitions of aphantasia exist—as discussed by Cavedon-Taylor (2022)—such as people who lack voluntary imagery (Zeman et al., 2015), lack both voluntary and involuntary imagery (Keogh and Pearson, 2018), and lack conscious imagery but not unconscious imagery (Nanay, 2021). Moreover, from a clinical perspective, there are neurological (Zeman et al., 2015, 2016) and psychogenic (de Vito and Bartolomeo, 2016) aphantasia. This study adopted Zeman’s definition that people with aphantasia people lack voluntary visual imagery. We discuss the definitions of aphantasia again in the General Discussion section.

### 1.1. Identification criteria and prevalence ratio of aphantasia

To identify people with aphantasia, most previous studies have adopted the Vividness of Visual Imagery Questionnaire (VVIQ: Marks, 1973). The VVIQ comprises of 16 items scored on a five-point Likert scale ranging from 16 to 80 points (with lower scores indicating weaker visual imagery vividness). Previous studies have used various ranges to identify aphantasia, such as VVIQ scores of 16 (Knight et al., 2022), 16–32 (Zeman et al., 2015; Keogh et al., 2018; Wicken et al., 2021; Dance et al., 2021b, 2022), 16–25 (Bainbridge et al., 2021; Pounder et al., 2022), and 16–23 (Zeman et al., 2020; Milton et al., 2021; Monzel et al., 2021b, 2023a,b; Monzel and Reuter, 2023; Wittmann and Şatırer, 2023). One previous study reported the proportion of people with aphantasia using a large sample (*N* = 1,004), including undergraduate students and the general population (Dance et al., 2022). They adopted a range of VVIQ ≤32 and demonstrated a prevalence ratio of 3.9%. This ratio included people with a complete lack of visual imagery (0.8%: VVIQ = 16) and dim or vague imagery (3.1%: 17 ≤ VVIQ ≤32).

Numerous studies have used VVIQ criteria to identify aphantasia. Furthermore, perceptual and cognitive experiments have been conducted to determine the characteristics of people with aphantasia, focusing on tasks associated with visual imagery. These tasks comprise the binocular rivalry paradigm (Keogh and Pearson, 2018), a memory task (Jacobs et al., 2018; Keogh et al., 2018; Monzel et al., 2021b; Knight et al., 2022; Wittmann and Şatırer, 2023), a visual search task (Monzel et al., 2021a; Monzel and Reuter, 2023), a sensory sensitivity task (Dance et al., 2021b), a neuropsychological test (Pounder et al., 2022), the recall of episodic and semantic memory details (Bainbridge et al., 2021), skin conductance level for the emotion behind imagery (Wicken et al., 2021), and pseudo-hallucinations (Königsmark et al., 2021).

Along with the VVIQ criterion, previous studies have also used the self-identification of a reduction in visual imagery based on individual statements or responses to questions from researchers (Jacobs et al., 2018; Keogh and Pearson, 2018; Königsmark et al., 2021; Dance et al., 2021a; Monzel et al., 2021a). However, no studies have examined the prevalence ratio of aphantasia using the self-identification of a reduction in visual imagery. The large sample (*N* = 2,500) used in a previous study (Faw, 2009) helped estimate the proportion of people who are aware of a reduction in visual imagery. However, this study did not aim to identify cases of aphantasia. This study proposed a single statement (i.e., “When you try to form a mental picture, it is usually: ‘no image,’ ‘vague and dim,’ ‘somewhat clear’ image, and ‘very clear’ image”) and obtained a prevalence ratio of 2.1% for “no image” and 8.2% for “vague and dim,” suggesting a ratio between 10 and 11% overall. Although the results (Faw, 2009) are informative, the prevalence ratio using self-identification of a reduction in visual imagery has not been calculated.

Typically, VVIQ is useful for rigorously defining aphantasia in an experimental setting. However, we hypothesize that self-identification may also be valid in some cases, such as during a preliminary broad survey. Furthermore, there may be a large discrepancy in the prevalence ratio between the criteria for the VVIQ and self-identification of a reduction in visual imagery. As these criteria have not been considered for the same participants, we do not know the extent of the discrepancy between them. To calculate the prevalence ratio of self-identification and examine the discrepancies between these criteria, this study conducted a direct comparison between the criteria for the VVIQ and self-identification by obtaining both sets of data from the same sample, though previous studies have already calculated the prevalence ratio for each criterion. This comprises our first research question.

### 1.2. Characteristics of aphantasia

Considering multisensory imagery, we should examine multiple types of sensory imagery modalities in people with aphantasia (e.g., auditory or tactile imagery) along with visual imagery. Moreover, cognitive style may be an important characteristic in people with aphantasia because people with aphantasia may tend to employ non-visual thinking strategies, such as the verbalizer type, if they exhibit a reduction in—or absence of—visual imagery. However, as the criteria for visual imagery (VVIQ or self-identification) have been used to define aphantasia, multisensory imagery and cognitive style have not been examined extensively. This constitutes our second research question.

#### 1.2.1. Multisensory imagery in people with aphantasia

Dawes et al. (2020) emphasized multisensory imagery’s importance in people with aphantasia. They used the Questionnaire upon Mental Imagery (QMI: Betts, 1909; shortened version: Sheehan, 1967), which measures the vividness of multisensory imagery, to investigate multisensory imagery’s characteristics in people with aphantasia; they identified people with aphantasia using self-identification of the absence of visual imagery (*N* = 317) and a low VVIQ score (VVIQ ≤32). Furthermore, they demonstrated that all sensory imagery in the QMI (i.e., visual, auditory, cutaneous, kinesthetic, gustatory, olfactory, and organic imagery) was weaker among people with aphantasia than among those without aphantasia. However, few studies have examined multisensory imagery in people with aphantasia, and further research is needed. Furthermore, we used the QMI to discuss multisensory imagery in people with aphantasia.

#### 1.2.2. Cognitive style in people with aphantasia

Almost no studies have examined the cognitive style of people with aphantasia. To measure cognitive style, Richardson (1977) focused on the visualizer (which predominantly uses visual thinking strategies) and verbalizer (which predominantly uses verbal thinking strategies) types of individuals and proposed the Verbalizer-Visualizer Questionnaire (VVQ). Using the VVQ, we can examine the verbalizer and visualizer types’ tendencies in people with aphantasia. If people with aphantasia cannot create visual imagery, they will find it difficult to think visually. Thus, a weak tendency toward visualization may exist in people with aphantasia. This point is discussed herein.

### 1.3. The current study

We conducted a large-sample investigation using online sampling (Study 1: 2,872 individuals of various ages and occupations from the general population) to investigate the proportion of people with aphantasia using the VVIQ and self-identified reduction in visual imagery criteria. First, participants with visual aphantasia were classified using the criteria primarily used in previous studies. After collecting responses based on the VVIQ and self-identification criteria from the same participants, we examined whether the proportions were consistent between the VVIQ and self-identification criteria. Although collective considerations may be advantageous, previous studies have calculated the proportions of each criterion (Faw, 2009; Dance et al., 2022). Consequently, we used the QMI and VVQ to evaluate the cognitive profiles of multisensory imagery and cognitive style, respectively, in participants with visual aphantasia defined by the criteria for VVIQ and self-identification. Findings for multi-sensory imagery (Dawes et al., 2020) were replicated by including detailed perspectives, and the findings for cognitive style were newly presented in this study. Furthermore, we focused on face recognition ability and conducted an additional investigation using online sampling (Study 2: 774 individuals) to examine the discrepancy in proportions between the criteria for the VVIQ and self-identification.

## 2. Study 1: appearance ratio and imagery properties of aphantasia

### 2.1. Materials and methods

#### 2.1.1. Participants

The participants included 2,900 Japanese individuals (1,452 males, 1,424 females, 5 others, and 19 no answer; mean age = 38.3 years, *SD* = 11.5), most of whom were recruited through online sampling (*n* = 2,657: 1,370 males, 1,266 females, 4 others, and 17 no answer; mean age = 39.8 years, *SD* = 10.7); the other participants were recruited through face-to-face sampling (*n* = 243: 82 males, 158 females, one other, and two with no answer; mean age = 22.7 years, *SD* = 8.2). Most participants were office workers, executives, or businesses owners (Table 1). The questionnaires were distributed to participants after they received a comprehensive explanation of the study and provided informed consent. All participants provided their consent to participate and responded anonymously. The ethics committee of Fukushima University approved the study protocol (approval number: 2021-01). This study was conducted in accordance with the Declaration of Helsinki and Ethical Guidelines for Medical and Biological Research Involving Human Subjects in Japan.

**Table 1:** Demographic and questionnaire data based on each criterion.

Measures criterion	VVIQ		Self-identification	Control
	(*n* = 105)		(*n* = 348)	(*n* = 2,465)
	With self-identification	Without self-identification		
	(*n* = 46)	(*n* = 59)	(*n* = 301)	
	VVIQ ≤ 32	VVIQ ≤ 32	VVIQ ≥ 33	VVIQ ≥ 33
Prevalence ratio	3.7%		12.1%	
Each group	1.6%	2.1%	10.5%	
Sample means (SD)				
Age (years)	40.67 (9.53)	35.29 (9.38)	39.78 (10.86)	38.20 (11.65)
Education (years)	14.52 (1.64)	14.75 (2.43)	14.78 (2.14)	14.81 (2.00)
Age awareness	24.83 (12.27)		25.82 (11.88)	
VVIQ full	27.26 (4.48)	29.08 (2.82)	43.64 (7.37)	48.72 (8.35)
F1: Relative or friend	6.65 (2.16)	7.98 (2.12)	10.13 (2.57)	12.64 (2.85)
F2: Rising sun	8.46 (1.94)	8.44 (2.25)	12.49 (2.58)	13.22 (2.58)
F3: Familiar shop	6.37 (1.80)	6.36 (1.67)	10.72 (2.79)	11.79 (2.75)
F4: Country scene	5.78 (1.38)	6.31 (1.45)	10.30 (2.67)	11.08 (2.90)
QMI full	113.57 (31.70)	136.98 (29.03)	158.08 (27.62)	174.34 (25.36)
Visual	13.33 (5.18)	16.88 (4.43)	18.92 (4.32)	22.96 (4.33)
Auditory	18.65 (6.23)	21.31 (5.28)	24.16 (4.99)	25.73 (4.42)
Cutaneous	14.13 (5.11)	19.63 (5.88)	21.12 (5.19)	23.77 (4.98)
Kinesthetic	17.57 (6.53)	20.90 (5.49)	24.14 (5.00)	25.98 (4.68)
Gustatory	18.02 (6.53)	21.29 (6.77)	24.45 (5.80)	26.96 (4.84)
Olfactory	13.83 (4.60)	17.25 (5.21)	20.88 (5.80)	22.74 (5.12)
Organic	18.04 (6.96)	19.73 (6.46)	24.40 (5.69)	26.20 (5.08)
VVQ verbalization	2.24 (1.82)	2.61 (1.45)	2.62 (1.70)	3.12 (1.77)
Visualization	2.43 (1.70)	2.80 (1.47)	2.79 (1.72)	3.75 (1.70)
Sample frequencies				
Sex ratio (M:F:Other:No)	26:18:0:2	28:31:0:0	192:107:0:2	1,193:1252:5:15
Profession				
Office worker, executive	13 (28.26%)	22 (37.29%)	111 (36.88%)	810 (32.86%)
Businesses owners	9 (19.57%)	6 (10.17%)	64 (21.26%)	410 (16.63%)
Professional, technical jobs	3 (6.52%)	1 (1.69%)	4 (1.33%)	84 (3.41%)
Public service worker	0 (0%)	0 (0%)	1 (0.33%)	17 (0.69%)
Student	1 (2.17%)	9 (15.25%)	18 (6.00%)	293 (11.89%)
Homemaker	3 (6.52%)	7 (11.86%)	25 (8.31%)	317 (12.86%)
Part-time job	7 (15.22%)	8 (13.56%)	34 (11.30%)	296 (12.01%)
Unemployed, retired	8 (17.39%)	6 (10.17%)	40 (13.29%)	186 (7.55%)
Others	2 (4.35%)	0 (0%)	4 (1.33%)	52 (2.11%)

M, Male; F, Female.

To avoid bias in participant recruitment (i.e., the participation of people with extremely high or low imagery), we excluded the term “aphantasia” from the survey. However, we used the term “imagery” to indicate the survey’s theme, which was required to ensure informed consent from the participants.

We used 2,871 participants (1,439 males, 1,408 females, 5 others, and 19 no answer; mean age = 38.3 years, *SD* = 11.5) in the analysis and excluded 29 participants who either provided incomplete responses or reported a psychiatric disorder. We aimed to investigate congenital aphantasia in participants; psychopathological factors may play a role in the context of acquired aphantasia (Monzel et al., 2022a). Thus, to distinguish congenital aphantasia from psychopathological factors (Zeman et al., 2016), we excluded participants who reported psychiatric disorders.

#### 2.1.2. Materials

##### 2.1.2.1. VVIQ

To examine the proportion of people with aphantasia based on visual imagery, we used the VVIQ (Japanese version: Hasegawa, 1993), which has been widely used to identify people with aphantasia. The VVIQ comprises four subfactors, including multiple situations, such as “relative and friend,” “rising sun,” “familiar shop,” and “country scene” (Kihlstrom et al., 1991). Each factor comprises four items evaluated on a five-point Likert scale from 1 (i.e., *no image at all, you only “know” that you are thinking of the object*) to 5 (i.e., *perfectly clear and as lively as seeing it for real*). Lower scores indicate weaker vividness of the visual imagery. The VVIQ’s Japanese version has been used in previous studies, and its validity and reliability have been confirmed (Hasegawa, 1993).

##### 2.1.2.2. Self-identification of reduction in visual imagery

We adopted the self-identification of reduction in visual imagery. We used a three-point Likert scale, based on scores of 1 (*I cannot imagine it at all*), 2 (*I can barely imagine it*), and 3 (*I can imagine it*), and asked the participants, “Can you create imagery, such as furniture in your room or a friend’s face?” We categorized people as having aphantasia if they scored 1 (i.e., “*I cannot imagine it at all*”) or 2 (i.e., “*I can barely imagine it*”) in this study.

If they answered, “*I can barely imagine it*” or “*I cannot imagine it at all*,” we asked 19 further questions (free descriptions) regarding their episodes, following Zeman et al. (2015); example questions are “When did you become aware that you could not form mental images?” and ‘Did it affect your career choice?’ We did not include these results herein because our aim was only to determine the proportion of participants with aphantasia.

##### 2.1.2.3. QMI

We adopted the QMI’s Japanese version (Tanabe and Hibino, 1986) to examine the cognitive profiles using multisensory imagery. The QMI comprises seven subfactors, including multiple sensory modalities—namely, “visual,” “auditory, “cutaneous,” “kinesthetic,” “gustatory,” “olfactory,” and “organic” imagery (Sheehan, 1967). Each factor includes five items evaluated on a seven-point Likert scale ranging from 1 (i.e., *no image at all, you only “know” that you are thinking of the object*) to 7 (i.e., *perfectly clear and as lively as seeing it for real*). Lower scores indicate weaker vividness of the sensory imagery. The QMI’s Japanese version has been used in previous studies and its validity and reliability have been confirmed (Watanabe et al., 2006).

##### 2.1.2.4. VVQ

We adopted the VVQ’s Japanese version (Hasegawa, 1993) to examine cognitive style (i.e., verbalization and visualization strategies). The VVQ, which comprises 15 items evaluated based on two choices (“*yes*” or “*no*”), was developed to analyze verbalizer and visualizer scales as either one-dimensional (Richardson, 1977) or two-dimensional factors (Kirby et al., 1988). Considering that the results of Kirby et al.’s (1988) factor analysis indicated the two-dimensional factor’s validity, we analyzed the verbalizing (items: 4, 7, 10, 14, 19, 22, and 25) and visualizing (items: 5, 12, 13, 20, 24, and 26) factors separately. The Japanese version of the QMI has been used in previous studies and its validity and reliability are confirmed (Hasegawa, 1993).

#### 2.1.3. Procedure

We administered the survey using Google Forms and paper-based questionnaires. Most participants (2,658 via online sampling and 192 of 242 via face-to-face sampling) completed the questionnaire on Google Forms, while only 50 participants from face-to-face sampling completed the paper-based questionnaire.

#### 2.1.4. Data analyses

To investigate the relationships among the QMI’s multisensory imagery and among the VVQ’s verbalizer and visualizer types, we adopted cluster analysis using Ward’s method (Ward, 1963). Cluster analysis is an analytical method for determining the coherence in multiple complex datasets, whereas Ward’s method determines the distance between clusters. First, the sums of squares were obtained for all data combinations. The first cluster was based on the smallest sum of squares. Thereafter, the sums of squares were calculated repeatedly for each combination included in that cluster and for the cluster creation process. Thus, we performed this technique because it helps us identify groups based on the sensory modalities of imagery or cognitive style.

### 2.2. Results and discussion

Table 1 presents the demographic data (i.e., age, years of education, age of awareness of the absence of visual imagery, sex ratio, and profession) and questionnaire results.

#### 2.2.1. Prevalence ratio of aphantasia

##### 2.2.1.1. VVIQ criteria

**Figure 1:**
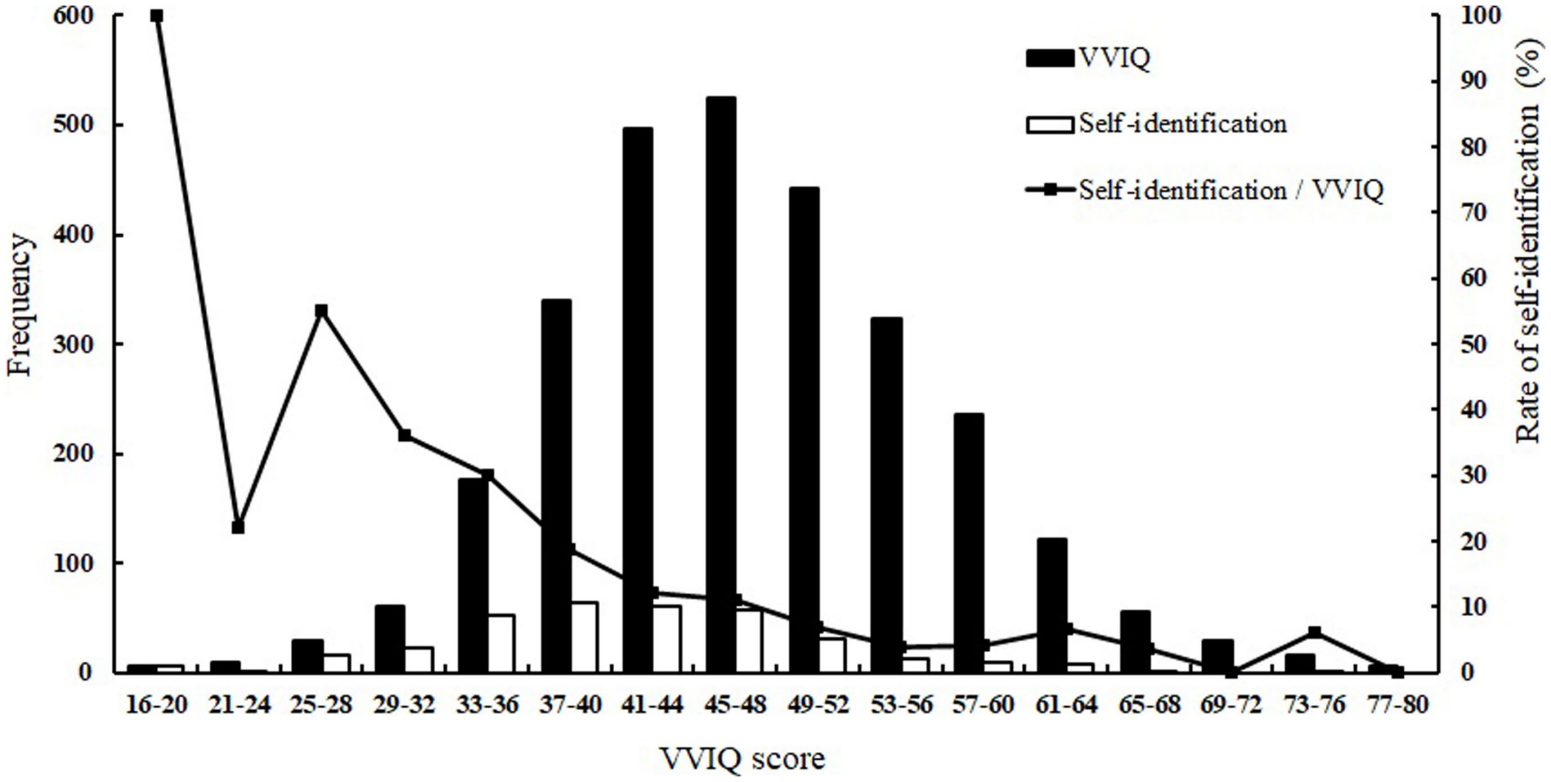
Frequencies and proportions of VVIQ and self-identification of the reduction in visual imagery. The black bar graph illustrates the frequencies for each VVIQ score (*n* = 2,871), the white bar graph illustrates the frequencies of self-identification (*n* = 301) in each VVIQ score, and the line graph shows the rate of self-identification for each VVIQ class.

Following Dance et al. (2022), we calculated the proportion of individuals who scored 16 (i.e., a complete absence of visual imagery) and 17–32 on the VVIQ. Consequently, we found proportions of 0.07% (i.e., 2 of 2,871 participants) and 3.6% (i.e., 103 of 2,871 participants), as illustrated in Table 1. Figure 1 (black bar) presents the VVIQ scores’ frequencies. The VVIQ frequency for each score was normally distributed, in the range of 16–80. However, the frequency significantly increased after 33.

These results indicate that approximately 3.7% of participants scored 16–32 on the VVIQ—in line with a previous study (Dance et al., 2022: 3.9%). However, the percentage of participants who scored 16 on the VVIQ (0.07%) was lower than that in the previous study (Dance et al., 2022: 0.8%).

##### 2.2.1.2. Self-identification of reduction in visual imagery

We defined people with aphantasia as those with a score of 1 (i.e., “*I cannot imagine it at all*”) or 2 (i.e., “*I can barely imagine it*”) for self-identification. As some people with aphantasia could create imagery for a moment, we included those with a score of 2.

We calculated the proportion of participants who reported a reduction in visual imagery. Figure 1 (white bar) presents the frequency of the self-identification of a reduction in visual imagery for each VVIQ score. The frequency of self-identification for each VVIQ score ranged from 16 to 76. When all the VVIQ scores were included, the proportion was 12.1% (i.e., 347 out of 2,871 participants), of which 0.6% (i.e., 16 of 2,871 participants) responded with 1 (i.e., “*I cannot imagine it at all*”) and 11.5% (i.e., 331 out of 2,871 participants) responded with 2 (i.e., “*I can barely imagine it*”).

These results indicate that approximately 12.1% of participants reported a reduction in visual imagery. These findings are similar to previously reported results (Faw, 2009: 10 to 11%).

#### 2.2.2. The discrepancy between criteria for VVIQ and self-identification

We found a discrepancy between the proportions of methods used for measuring visual imagery. Figure 1 (line graph) illustrates the self-identification rate for each VVIQ class. Higher rates would result from consistent proportions of the two measures for VVIQ and self-identification, particularly for VVIQ scores rating from 16 to 32, which exhibited rates of 22.22–100%. We classified the participants into four groups by combining the VVIQ and self-identification questionnaires’ results (Table 1). The participants (*n* = 46) reported that they could not form visual imagery under the self-identification criterion, responding with 1 (i.e., “*I cannot imagine it at all*”) or 2 (i.e., “*I can barely imagine it*”) and exhibited a low VVIQ score (VVIQ ≤32). However, some participants (*n* = 59) reported that they could form visual imagery under the self-identification criterion, responding with 3 (i.e., “*I can imagine it*”) despite exhibiting a low VVIQ score (VVIQ ≤32). Moreover, for VVIQ scores ≥33, some self-identification frequency was observed. These participants (*n* = 301) reported that they could not form visual imagery in the self-identification measure (1 or 2), despite exhibiting a high VVIQ score (VVIQ ≥33). By contrast, other participants (*n* = 2,465) reported that they could form visual imagery in the self-identification measure (3) and exhibited a high VVIQ score (VVIQ ≥33). Study 2 examines these discrepancies in detail.

#### 2.2.3. Characteristics of aphantasia

We used the criteria for VVIQ (VVIQ ≤32) and self-identification, and divided these individuals into three groups—specifically, an aphantasia group (VVIQ ≤32) and two control groups with and without self-identification. We considered that a low VVIQ (VVIQ ≤32) might indicate aphantasia, as proposed in several previous studies (Zeman et al., 2015; Dance et al., 2022). Moreover, although previous studies suggest that groups with VVIQ ≥33—with and without self-identification—should be used as a single control group, we treated them as separate control groups owing to the variations observed in self-identification.

##### 2.2.3.1. Multisensory imagery

We examined the cognitive profiles of multisensory imagery in the QMI to investigate the relationship between visual imagery and other sensory imagery in people with and without visual aphantasia, as defined by the VVIQ and self-identification criteria. We calculated the z-score using the mean scores of each subfactor (i.e., seven modalities) in the QMI and performed a cluster analysis (Ward’s method) for each group. We classified the clusters using distance <10 in the dendrogram for each group.

**Figure 2:**
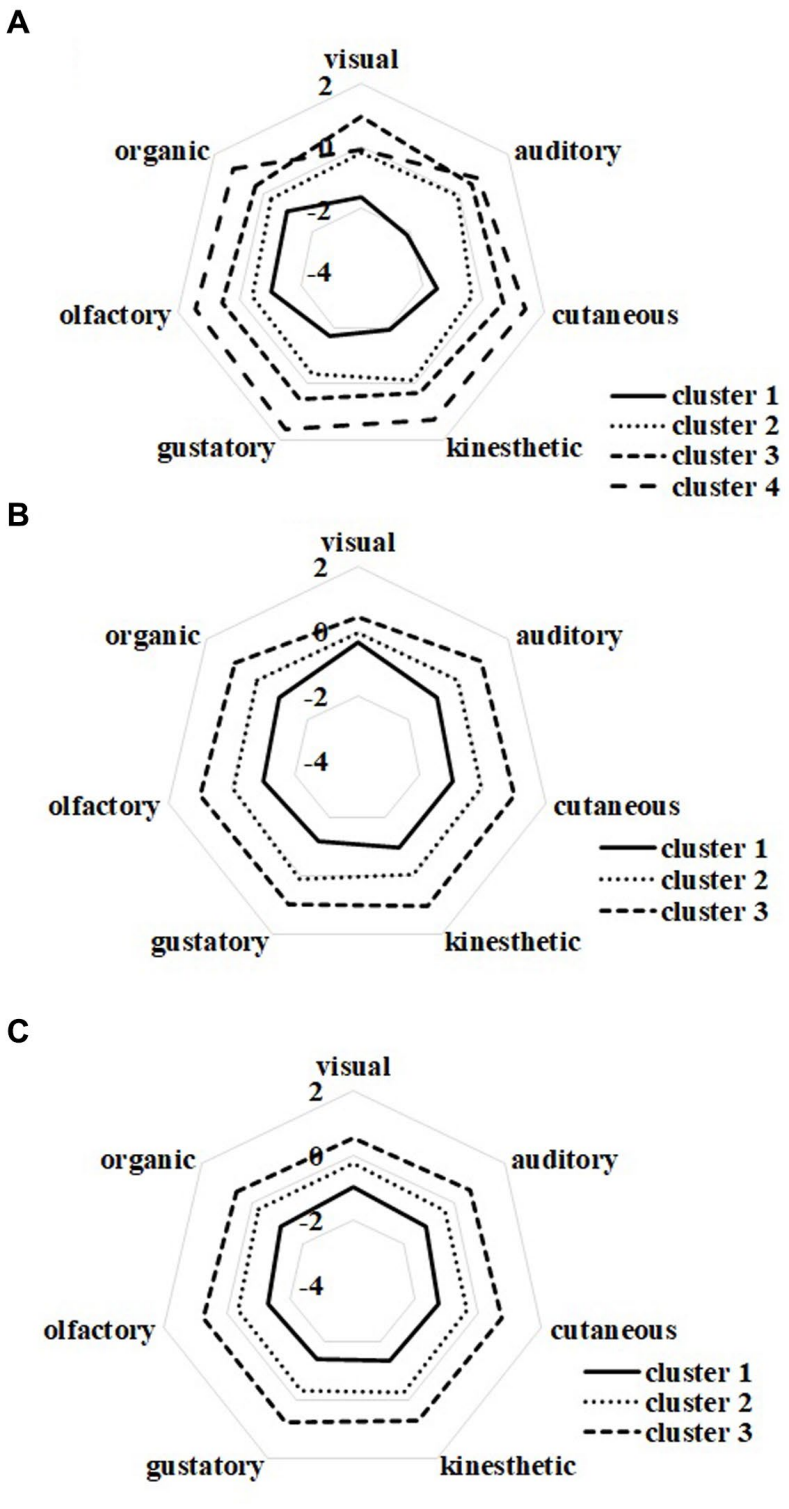
Cognitive profiles for multisensory imagery. **(A–C)** Show aphantasia and control groups with and without self-identification, respectively.

The cluster analysis indicated that the participants in the aphantasia group could be classified into four clusters (Figure 2A). Subsequently, we examined the differences between clusters for each sensory modality of imagery. Significant differences indicated that a cluster was severely unable to create imagery in that sensory modality, suggesting individual differences in imagery formation. Furthermore, the significant differences found only in some modalities may represent a specificity in the sensory modality of imagery. We performed a one-way analysis of variance (ANOVA) of QMI scores using clusters as a between-participants factor. We found the main effects of clusters in all modalities (visual: *F*[3, 101] = 34.27, *p* < 0.001, η*p^2^* = 0.50; auditory: *F*[3, 101] = 37.76, *p* < 0.001, η*p^2^* = 0.53; cutaneous: *F*[3, 101] = 49.69, *p* < 0.001, η*p^2^* = 0.60; kinesthetic: *F*[3, 101] = 38.21, *p* < 0.001, η*p^2^* = 0.53; gustatory: *F*[3, 101] = 64.78, *p* < 0.001, η*p^2^* = 0.66; olfactory: *F*[3, 101] = 34.67, *p* < 0.001, η*p^2^* = 0.51; organic: *F*[3, 101] = 15.92, *p* < 0.001, η*p^2^* = 0.32). A multiple comparison analysis using the Bonferroni correction (Supplementary Table A1) revealed that the z-scores were larger in the order of clusters 1–4, for most of the auditory, cutaneous, kinesthetic, gustatory, olfactory, and organic imagery (−12.39 < *t* < −2.90, 0.001 < *p* < 0.027); however, no significant differences were found between clusters 3 and 4 for auditory (*t* = −1.21, *n.s.*), and clusters 1 and 2 for olfactory (*t* = −2.33, *n.s.*) and organic (*t* = −2.22, *n.s.*) imagery. Regarding visual imagery, cluster 1’s z-score was the lowest, and cluster 3’s z-score was the highest (−9.42 < *t* < 4.15, *p* < 0.001), but we found no significant difference between clusters 2 and 4 (*t* = −0.42, *n.s.*).

In the control group with self-identification, the cluster analysis revealed that the participants could be classified into three clusters (Figure 2B). We performed a one-way ANOVA using clusters as the between-participants factor and found main effects in all modalities (visual: *F*[2, 298] = 13.72, *p* < 0.001, η*p^2^* = 0.08; auditory: *F*[2, 298] = 103.92, *p* < 0.001, η*p^2^* = 0.41; cutaneous: *F*[2, 298] = 154.71, *p* < 0.001, η*p^2^* = 0.51; kinesthetic: *F*[2, 298] = 165.77, *p* < 0.001, η*p^2^* = 0.53; gustatory: *F*[2, 298] = 223.77, *p* < 0.001, η*p^2^* = 0.60; olfactory: *F*[2, 298] = 168.59, *p* < 0.001, η*p^2^* = 0.53; organic: *F*[2, 298] = 98.48, *p* < 0.001, η*p^2^* = 0.40). A multiple comparison analysis using Bonferroni correction (Supplementary Table A2) revealed that the z-scores were lower in the order of clusters 1, 2, and 3 in cutaneous, kinesthetic, gustatory, olfactory, and organic imagery (−21.06 < *t* < −7.40, *p* < 0.001). For visual imagery, we found that the z-scores for cluster 3 were higher than that for cluster 1 (*t* = −5.10, *p* < 0.001) and that for cluster 2 (*t* = −3.80, *p* < 0.005), but there was no significant difference between clusters 1 and 2 (*t* = −2.00, *n.s.*).

In the control group without self-identification, cluster analysis revealed that the participants could be classified into three clusters (Figure 2C). We performed a one-way ANOVA using clusters as the between-participants factor and found their main effects in all modalities (visual: *F*[2, 2462] = 459.38, *p* < 0.001, η*p^2^* = 0.27; auditory: *F*[2, 2462] = 789.46, *p* < 0.001, η*p^2^* = 0.39; cutaneous: *F*[2, 2462] = 1168.77, *p* < 0.001, η*p^2^* = 0.49; kinesthetic: *F*[2, 2462] = 1091.15, *p* < 0.001, η*p^2^* = 0.47; gustatory: *F*[2, 2462] = 1368.67, *p* < 0.001, η*p^2^* = 0.53; olfactory: *F*[2, 2462] = 1196.78, *p* < 0.001, η*p^2^* = 0.49; organic: *F*[2, 2462] = 666.26, *p* < 0.001, η*p^2^* = 0.35). A multiple comparison analysis using Bonferroni correction (Supplementary Table A3) revealed that the z-scores were lower in the order of clusters 1, 2, and 3 in all modalities (−47.48 < *t* < −13.53, *p* < 0.001).

Collectively, multiple profile types were identified in the aphantasia group. Some participants scored low for all sensory modalities, whereas others scored showed low for visual imagery only. However, no significant differences were found between clusters in visual imagery (i.e., no significant differences existed between clusters 2 and 4), though the scores for other type of sensory imagery had a similar profile. This unique tendency toward a reduction in visual imagery was not observed in the control groups with and without self-identification.

##### 2.2.3.2. Cognitive style

We examined cognitive style using the VVQ in people with and without visual aphantasia, as defined by the VVIQ and self-identification criteria. We calculated the z-score using the mean scores of each subfactor (i.e., verbalizer and visualizer types) in the VVQ and performed a cluster analysis (Ward’s method) for each group. We classified the clusters using distance <10 in the dendrogram for each group.

**Figure 3:**
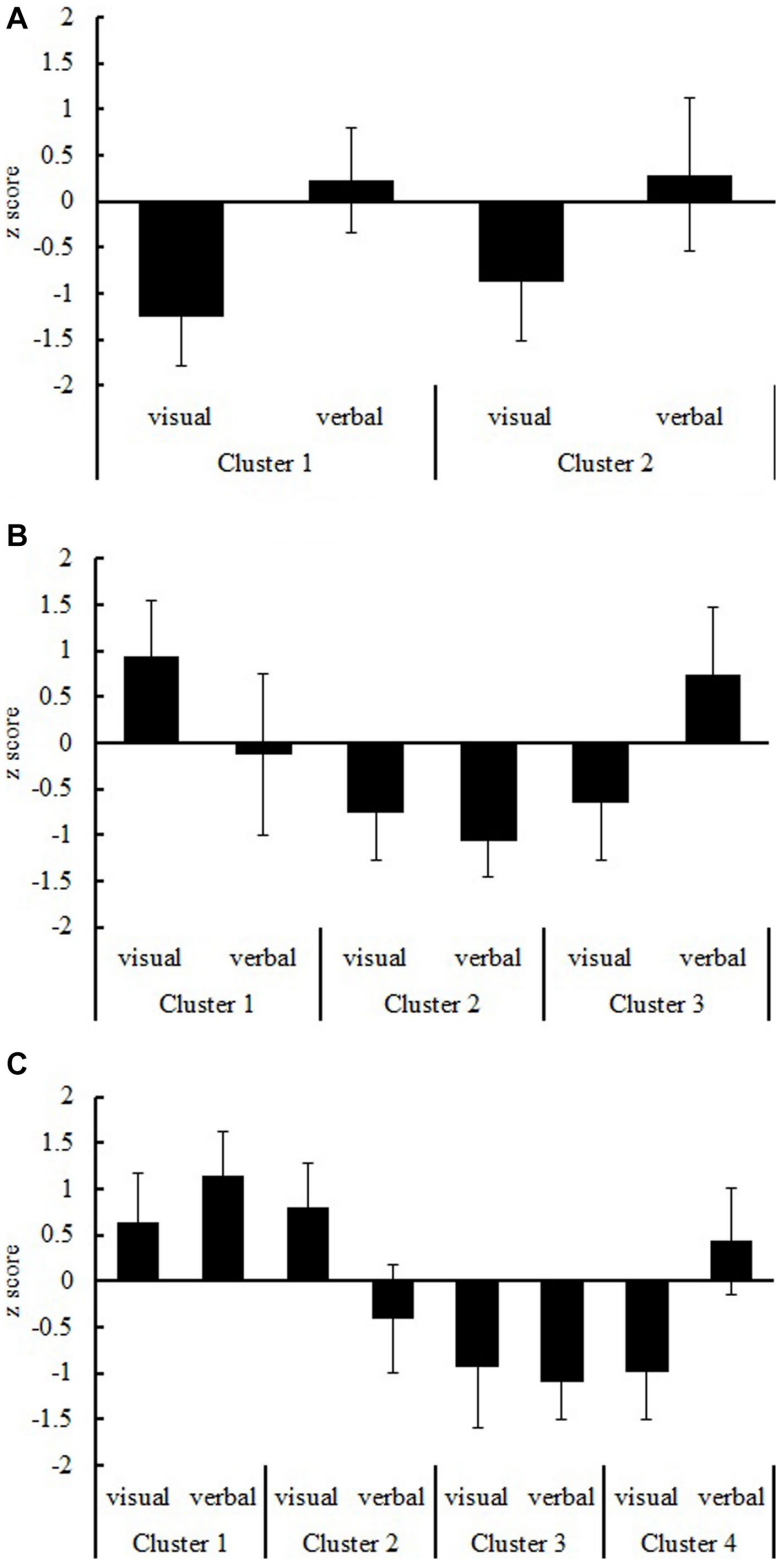
Cognitive styles for the visualizer and verbalizer types. **(A–C)** Show aphantasia and control groups with and without self-identification, respectively.

The cluster analysis indicated that the participants in the aphantasia group could be classified into two clusters (Figure 3A). To examine the relationship between the verbalizer and visualizer factors in these two clusters, we conducted a *t*-test using clusters as a between-participants factor. In both the verbalizer and visualizer factors, no significant differences were observed in the z-scores between clusters 1 and 2 (verbalizer: *t*[103] = −0.19, *n.s.*, Cohen’s *d* = −0.04; visualizer: *t*[103] = −1.12, *n.s.*, Cohen’s *d* = −0.22).

In the control group with self-identification, cluster analysis revealed that the participants could be classified into three clusters (Figure 3B). We performed a one-way ANOVA using clusters as the between-participants factor and found main effects for both the verbalizer and visualizer factors (verbalizer: *F*[2, 298] = 119.25, *p* < 0.001, η*p^2^* = 0.45; visualizer: *F*[2, 298] = 269.91, *p* < 0.001, η*p^2^* = 0.64). A multiple comparison analysis using Bonferroni correction revealed that the z-scores were higher in the order of clusters 1, 3, and 2 in the verbalizer factor (clusters 1 vs. 2: *t*[298] = 8.03, *p* < 0.001, Cohen’s *d* = 1.24; clusters 1 vs. 3: *t*[298] = −8.99, *p* < 0.001, Cohen’s *d* = −1.16; clusters 2 vs. 3: *t*[298] = −15.22, *p* < 0.001, Cohen’s *d* = −2.40). For the visualizer factor, the z-scores were significantly higher for cluster 1 than for clusters 2 and 3 (clusters 1 vs. 2: *t*[298] = 18.21, *p* < 0.001, Cohen’s *d* = 2.81; clusters 1 vs. 3: *t*[298] = 20.41, *p* < 0.001, Cohen’s *d* = 2.64); however, no significant difference was observed between the z-scores for clusters 2 and 3 (*t*[298] = −1.11, *n.s.*, Cohen’s *d* = −0.18).

In the control group without self-identification, the cluster analysis revealed that the participants could be classified into four clusters (Figure 3C). We performed a one-way ANOVA using clusters as the between-participants factor and found main effects for both the verbalizer and visualizer factors (verbalizer: *F*[3, 2461] = 2257.51, *p* < 0.001, η*p^2^* = 0.73; visualizer: *F*[3, 2461] = 1838.93, *p* < 0.001, η*p^2^* = 0.69). A multiple comparison analysis using Bonferroni correction (Supplementary Table B1) indicated that the z-scores were lower in the order of clusters 1, 4, 2, and 3 in the verbalizer factor (−47.52 < *t* < −76.58, *p* < 0.001). In the visualizer factor, the z-score of cluster 2 was higher than that of cluster 1, and these z-scores were higher than those of clusters 3 and 4 (−6.01 < *t* < 56.41, *p* < 0.001). No difference was observed between the z-scores of clusters 3 and 4 (*t* = −1.45, *n.s.*).

Collectively, the aphantasia group exhibited non-specific characteristics pertaining to cognitive style (verbalizer and visualizer types), though the control group with self-identification exhibited verbalizer and visualizer types, and the control group without self-identification exhibited verbalizer, visualizer, and mixed types.

#### 2.2.4. Correlations among questionnaires

We conducted a correlation analysis among all questionnaires in the three groups. In the aphantasia group (Supplementary Table C1), we observed significant correlations among the VVIQ subfactors (*r* = −0.198), among the QMI subfactors (0.235 ≤ *r* ≤ 0.733), and among the VVQ subfactors (*r* = 0.511). Moreover, we found significant correlations between the QMI and VVIQ subfactors (0.202 ≤ *r* ≤ 0.503), QMI and VVQ subfactors (0.213 ≤ *r* ≤ 0.255), and VVIQ and VVQ subfactors (0.249 ≤ *r* ≤ 0.264).

In the control group with self-identification (Supplementary Table C2), we found significant correlations among the VVIQ subfactors (0.182 ≤ *r* ≤ 0.767), QMI subfactors (0.220 ≤ *r* ≤ 0.640), and VVQ subfactors (*r* = 0.330); and between the QMI and VVIQ subfactors (0.127 ≤ *r* ≤ 0.610), QMI and VVQ subfactors (0.120 ≤ *r* ≤ 0.194), and VVIQ and VVQ subfactors (0.114 ≤ *r* ≤ 0.161).

In the control group without self-identification (Supplementary Table C3), we found significant correlations among the VVIQ subfactors (0.182 ≤ *r* ≤ 0.513), QMI subfactors (0.220 ≤ *r* ≤ 0.640), and VVQ subfactors (*r* = 0.330); and between the QMI and VVIQ subfactors (0.127 ≤ *r* ≤ 0.610), QMI and VVQ subfactors (0.120 ≤ *r* ≤ 0.194), and VVIQ and VVQ subfactors (0.114 ≤ *r* ≤ 0.161).

Significant correlations were found for most questionnaires in all groups. However, no significant correlations were found among the VVIQ subfactors in the aphantasia group. Furthermore, these participants might have provided disparate answers unlike the other participants who responded consistently.

## 3. Study 2: face recognition ability

In Study 2, we explored the factor pertaining to discrepancies in proportions between the criteria for the VVIQ and self-identification. We confirmed free descriptions reported by those who answered, “*I can barely imagine it*” or “*I cannot imagine it at all*,” to assess their self-identified visual imagery deficit. Specifically, we considered the following question; “How did you realize that you could not visualize?” Notably, the responses were related to a difficulty in face recognition or face imagery abilities, such as “I cannot visualize face” or “I cannot remember faces, so I cannot match the faces and names of people I meet at work” in the aphantasia group (12.8%) and self-identification group (22.9%). Thus, a certain number of individuals in each group had poor face recognition ability, including face imagery.

Considering the VVIQ and self-identification items’ contents, the VVIQ did not include any face-imagery question, but the self-identification measure did. Even if the participants scored high on the VVIQ (VVIQ ≥33), if they were aware of a reduction in face imagery during self-identification, they might be judged as the control group with self-identification. We speculate that some participants in the control group with self-identification might have had poor face recognition ability or face imagery. Therefore, face recognition ability might be one of the factors contributing to the discrepancy between the criteria for the VVIQ and self-identification. In Study 2, we administered a novel questionnaire survey and compared face recognition abilities among the groups.

### 3.1. Materials and methods

#### 3.1.1. Participants

Participants included 816 Japanese individuals (379 males, 435 females, and 2 others; mean age = 40.8 years, *SD* = 10.6) who did not participate in Study 1. Most participants were office workers, executives, or businesses owners. Questionnaires were distributed to participants after they received a comprehensive explanation of the study and provided informed consent. Participants consented to participate in the study and responded anonymously. The ethics committee of Fukushima University approved the study protocol (approval number: 2021-01). This study was conducted in accordance with the Declaration of Helsinki and Ethical Guidelines for Medical and Biological Research Involving Human Subjects in Japan.

#### 3.1.2. Materials

As in Study 1, we administered the VVIQ; and self-identification of a reduction in visual imagery, QMI, and VVQ. Additionally, we used the 20-item Prosopagnosia Index (PI20; Shah et al., 2015) to measure face recognition ability. We adopted the PI20’s Japanese version (PI20-J: Nakashima et al., 2020). The PI20-J comprises of a one-factor model containing 20 items evaluated on a five-point Likert scale ranging from 1 (i.e., *not at all applicable*) to 5 (i.e., *applicable*). Higher scores indicate a lower ability for face recognition. The PI20-J’s validity and reliability have been confirmed (Nakashima et al., 2020). Although the PI20-J measures prosopagnosia, it also includes items related to face recognition ability; thus, we adopted this questionnaire.

#### 3.1.3. Procedure

As in Study 1, we administered the survey using Google Forms. All participants completed the questionnaire on Google Forms, including the QMI, VVIQ, VVQ, PI20-J, and self-identification of a reduction in visual imagery, in that order.

### 3.2. Results and discussion

We used the data of 774 participants (351 males, 421 females, and 2 with no answers; mean age = 40.8 years, *SD* = 10.6) in the analysis and excluded 42 participants who provided incomplete responses.

We calculated the mean (*SD*) of the total score of PI20-J in each group: The aphantasia group, control group with self-identification, and control group without self-identification scored 59.80 (14.05), 63.13 (13.76), and 51.55 (14.42), respectively.

#### 3.2.1. Prevalence ratio of aphantasia in visual imagery scale

The proportions of people with aphantasia using the criteria for the VVIQ and self-identification of a reduction in visual imagery were 2.6 and 10.6%, respectively. The proportion calculated by the VVIQ seemed slightly lower than that in Study 1 (3.7%) and a previous study (3.9%: Dance et al., 2022), but it was within the range hypothesized in prior research (2 to 4%: Zeman et al., 2015). The proportion calculated by self-identification replicated the previous study’s fiding (Faw, 2009).

#### 3.2.2. Individual differences in face recognition ability

We performed a correlation analysis between the VVIQ and PI20-J scores in the three groups. If the correlation coefficient is higher in the control group with self-identification than in the aphantasia group, the larger proportion of VVIQ scores in the self-identification group is attributable to face recognition ability. If so, it can be demonstrated that the self-awareness of reduced visual imagery in the control group with self-identification is affected by face recognition ability.

In the aphantasia group, we observed no significant correlation (*r* = 0.271, *n.s.*), whereas we found a significant moderate correlation in the control group with self-identification (*r* = −0.412, *p* < 0.01) and a significant weak correlation in the control group without self-identification (*r* = −0.187, *p* < 0.01).

No significant correlation was found between the VVIQ and PI20-J in the aphantasia group, though a significant correlation existed between them in the control group with and without self-identification. Thus, comparing the aphantasia and control groups with self-identification, we assume that the VVIQ of the control group with self-identification is attributable to face recognition ability, and that the self-awareness of reduced visual imagery reported in the control group with self-identification might be affected by face recognition ability.

#### 3.2.3. Relationship between aphantasia and face recognition ability

We further examined the relationship between aphantasia and face recognition ability (PI20-J). Burns et al. (2022) summarized the findings pertaining to PI20, using 65 as the cutoff point for prosopagnosia. Shah et al. (2015) examined individuals who were suspected of having developmental prosopagnosia and administered questionnaires and objective tests, suggesting that PI20 scores of 65–74, 75–84, and 85–100 may indicate mild, moderate, and severe developmental prosopagnosia, respectively. Considering these results, we adopted a cut-off point of 65.

Based on this cutoff point, we divided our participants (Table 2) as follows: The aphantasia group had 20 people (8 people with PI20-J ≥ 65, 12 people with PI20-J ≤ 64), the control group with self-identification had 72 people (38 people with PI20-J ≥ 65, 34 people with PI20-J ≤ 64), and the control group without self-identification had 682 people (139 people with PI20-J ≥ 65, 543 people with PI20-J ≤ 64).

**Table 2:** Cross-tabulation of aphantasia and face recognition ability.

		Aphantasia		
		Aphantasia	Control group with self-identification	Control group without self-identification
Face recognition ability	PI20-J ≥ 65	8 (40.0%) (1.7)	38 (52.8%) (6.0)	139 (20.4%) (−6.3)
	PI20-J ≤ 64	12 (60.0%) (−1.7)	34 (47.2%) (−6.0)	543 (79.6%) (6.3)
Total		20	72	682

The upper value shows the number of people and ratio (in brackets), while the lower value shows the adjusted coefficient in the residual analysis. The aphantasia group includes four people with PI20-J ≥ 65 and six people with PI20-J ≤ 64.

In the cross-tabulation, we performed a chi-square test and residual analysis (Table 2). The significance level was set at *p* < 0.05. In the residual analysis after the chi-square test, the adjusted coefficient (±1.96) was used as a criterion of significant difference.

If an association exists between aphantasia and face recognition ability (PI20-J), there may be significant differences in the aphantasia group. A chi-square test revealed that aphantasia was significantly associated with lower face recognition ability (*χ*^2^ = 40.50). In the residual analysis, significant differences were observed in the control groups with and without self-identification, but not in the aphantasia group.

To directly examine the association between aphantasia and face recognition ability (PI20-J) in the aphantasia group, a chi-square test was performed again only in the aphantasia group. The results revealed no significant difference (*χ*^2^ = 2.09, *n.s.*). Thus, an association was found between aphantasia and face recognition ability, thus reflecting the self-identification and control groups’ results.

## 4. General discussion

This large-sample (*N* = 2,871) study examined the proportion of people with aphantasia in terms of visual imagery criteria using the VVIQ and self-identification of a reduction in visual imagery. Our data revealed a ratio of 3.7% for the VVIQ criterion (VVIQ ≤32) and 12.1% for the self-identification criterion. However, a large discrepancy was found between these proportions, whereby some participants reported self-identification of a reduction in visual imagery despite exhibiting VVIQ ≥33. Our additional questionnaire research suggested that this discrepancy may be caused by individual differences in face recognition ability. Moreover, we examined the imagery properties of people with aphantasia in terms of their multisensory imagery (QMI) and cognitive style (VVQ) profiles. For cognitive profiles of multisensory imagery, we found that some individuals lacked all sensory imagery, whereas others lacked the specific capacity for visual imagery, despite their ability to form all other types of sensory imagery. Regarding cognitive style, although we tried identifying verbalizer or visualizer types in people with aphantasia, these types were unclear in people with aphantasia.

### 4.1. Prevalence ratio of aphantasia

Our first aim was investigating the proportion of people with aphantasia using the VVIQ and self-identification in the absence of visual imagery. Thereafter, we replicated the proportion of 3.9% under VVIQ ≤32 (Dance et al., 2022) and 10 to 11% under self-identification (Faw, 2009), yielding results of 3.7 and 12.1%, respectively. In contrast to previous studies, this study included a larger number of participants (*N* = 1,004: Dance et al., 2022; *N* = 2,500: Faw, 2009; *N* = 715: Dawes et al., 2020). Moreover, our participants were primarily from the general population with diverse occupations, in contrast to previous studies that predominantly included students. Therefore, our study is more representative of the general population than prior students. However, all our participants were Japanese, and thus, cultural differences must be acknowledged.

### 4.2. Discrepancy between criteria for VVIQ and self-identification

We found a discrepancy between the VVIQ (3.7%) and self-identification of a reduction in visual imagery (12.1%). How can we explain the discrepancy between the proportions of VVIQ and self-identification criterion? Certainly, the 12.1% with self-identification included 3.7% with low VVIQ (VVIQ ≤32), but what were the imagery characteristics of the remaining 8%? This discrepancy is attributable to the absence of face recognition ability based on free descriptions.

Study 2’s results revealed face recognition ability as a possible factor contributing to this discrepancy. We assessed the relationship between VVIQ and face recognition ability using correlation analysis in each aphantasia group (VVIQ ≤32) and the control groups with and without self-identification (VVIQ ≥33). If the correlation coefficient is higher in the control group with self-identification than in the aphantasia group, the higher coefficient of the VVIQ in the control group with self-identification can be explained by face recognition ability. Based on this, we predicted that self-awareness of a reduction in visual imagery in the control group with self-identification depends on face recognition ability. A significant moderate correlation existed in the control group with self-identification and a significant weak correlation existed in the control group without self-identification, but not in the aphantasia group. Thus, we assume that face recognition ability was a factor causing this discrepancy.

However, one key point should be noted. The discrepancy could be an artifact resulting from the participants’ attention to “a friend’s face” in the questionnaire on self-identification. In other words, they may have responded only to the item regarding whether they could imagine the faces of their friends. If so, our present study included participants who, in fact, do not have a deficit in visual imagery but instead have a deficit in face recognition. Therefore, we believe that self-identification questions should be modified to measure the ability to form general object imagery (e.g., furniture, fruits, or cars) rather than predominantly face imagery. This might reduce the discrepancy between the VVIQ and self-identification.

One might argue that while the VVIQ may be a strict measure for identifying people with aphantasia, self-identification is not. Indeed, self-identification may be associated with a higher risk of false positives. Hence, our argument does not imply that VVIQ is unsuitable or that self-identification is preferable; instead, we propose using them according to the given purpose. Furthermore, numerous participants experienced problems during the interviews because they were aware of a reduction in visual imagery. To support these individuals, a broad and easy extraction is necessary. It can be used as a preliminary step prior to the VVIQ as a simple screening tool; we believe that the VVIQ should only be administered to those who agree to it as a research study. For example, a person who suspects that they may lack visual imagery would perform this simple screening. Alternatively, if a child has learning difficulties owing to a reduction in visual imagery, the questionnaire can first be administered in a simplified manner at school. Assumedly, these individuals will contact the researcher to conduct the VVIQ. As has already been highlighted, the self-identification question’s content should be carefully examined. The VVIQ is appropriate for recruiting participants for an experiment, whereas the self-identification method may help easily detect a wide range of individuals challenged by a reduction in visual imagery.

### 4.3. Characteristics of aphantasia

Our second aim was investigating the imagery properties of aphantasia by focusing on the cognitive profiles of multisensory imagery using the QMI and cognitive style (verbalizer or visualizer types) using the VVQ.

#### 4.3.1. Multisensory imagery

For cognitive profiles of multisensory imagery, while a previous study had already used QMI and examined multisensory imagery (Dawes et al., 2020), we used a larger sample in our study. Moreover, our participants were characterized based on a combination of the VVIQ and self-identification. Therefore, we examined multisensory imagery using multiple visual imagery tools. Consequently, we found that some participants exhibited a reduction in all sensory imagery, whereas others only lacked visual imagery and could form other types of sensory imagery, as proposed by Dawes et al. (2020). The former and latter participants may be interpreted as having multisensory and visual aphantasia (Monzel et al., 2022b), respectively. Monzel et al. (2022b) suggested various types of aphantasia focusing on multisensory imagery, including “visual aphantasia,” “auditory aphantasia” (see also “anauralia”: Hinwar and Lambert, 2021), and “multisensory aphantasia” (see also “dysikonesia”: Dance et al., 2021b).

Along with the visual imagery criteria for the VVIQ and self-identification, we used QMI and observed both visual and multisensory aphantasia. However, we would have been unable to distinguish multisensory aphantasia from visual aphantasia if we had used only the VVIQ. This indicates that visual aphantasia—as defined by the VVIQ—includes both visual and multisensory aphantasia. Notably, we are not opposed to using only visual criteria such as VVIQ to define visual aphantasia. However, we suggest that using visual and multisensory criteria can distinguish visual and multisensory aphantasia. Similarly, previous studies (Dawes et al., 2020; Zeman et al., 2020; Dance et al., 2021b) have demonstrated multisensory aphantasia (dysikonesia: Dance et al., 2021b). Specifically, people with aphantasia exhibit a reduction in visual imagery, as well as other sensory imagery (Dawes et al., 2020). Moreover, half (54.2%) people with aphantasia exhibit a reduction in imagery in any modality, in addition to visual imagery (Zeman et al., 2020). However, the present study only extracted visual and multisensory aphantasia. As imagery is multisensory, we believe that it is necessary to not only focus on visual imagery but also show aphantasia pertaining to auditory or other modalities in the absence of imagery. Therefore, we examined the differences in imagery modalities to explore the subtypes of aphantasia.

A similar observation may be made for auditory or other sensory types of aphantasia. For example, if we only use auditory criteria to identify auditory aphantasia (anauralia: Hinwar and Lambert, 2021), we can overlook the combinations of auditory and multisensory aphantasia. Therefore, each subtype of aphantasia or multisensory aphantasia must be classified using the criteria for multisensory imagery.

#### 4.3.2. Cognitive style

Almost no previous study has reported on the cognitive styles of people with aphantasia. We adopted the VVQ as a questionnaire to measure cognitive styles, such as verbalizer or visualizer types, and found nonspecific characteristics in the aphantasia group. We predicted that people with aphantasia would be less likely to be the visualizer type because of their weak visual imagery, which was found to be true. As control groups showed people with verbalizer and visualizer types, we assume that the lack of clarity between the verbalizer and visualizer types is a characteristic of aphantasia. This may be because the items determining the visualizer type are a combination of those that require visual imagery and those that do not, which may have precipitated the disjointed responses in people with aphantasia.

Another possibility is that the cognitive style pertaining to aphantasia cannot be adequately examined in terms of the verbalizer and visualizer types. Most previous studies have examined cognitive style in the visual-verbal dimension; similarly, we have also adopted this dimension. Moreover, Blajenkova et al. (2006) indicated the importance of object imagery (object properties of visual processing, including shape, color, and texture) and spatial imagery (spatial properties of visual processing, including object location, movement, and spatial relationships) in the visual dimension. Accordingly, they proposed the Object and Spatial Imagery Questionnaire (OSIQ), which measures object and spatial imagery as a visual dimension. From this perspective, Palermo et al. (2022) indicated that object aphantasia is associated with difficulties in imaging single items and events, and spatial aphantasia is associated with difficulties in spatial imagery and sense of direction. However, the OSIQ can only measure the visual dimension, such as object and spatial imagery, not the verbal dimension. For example, as a related questionnaire, the Object-Spatial Imagery and Verbal Questionnaire (OSIVQ: Blazhenkova and Kozhevnikov, 2009) measures both the visual dimension, including object and spatial imagery, and the verbal dimension; examining this questionnaire may enable a broader discussion of the cognitive style of aphantasia.

### 4.4. Limitations and future directions

Despite indicating a discrepancy between the criteria for the VVIQ and self-identification, one might argue that participants’ attention to the questionnaires might have affected the discrepancy. Participants may have provided different responses to the VVIQ and self-identification if they had not completed in the questionnaire. Moreover, we already highlighted that the participants might predominantly attend to “a friend’s face” in the self-identification question, causing artifact results of the discrepancy between the VVIQ and self-identification. Although we could not examine this possibility further because we did not perform any analysis pertaining to attention assessing the participants’ attention levels—particularly for an online sampling study comprising many participants—may be necessary.

Moreover, to underscore the discrepancy between these criteria, we asked participants to create an image of the furniture in their room and a friend’s face in the questionnaire using the self-identification criterion. A possibility exists that the discrepancy is a matter (Zeman et al., 2020) of “imagery” and “imagination.” Specifically, the people without aphantasia might have responded by interpreting “imagery” when asked to “imagine.” Similarly, people with aphantasia might have responded to the question by interpreting “imagination” when asked to “imagine.” Consequently, some people with aphantasia might have responded using “*I can imagine it*.” Thus, despite lacking imagery (furniture in a room and a friend’s face), they could know or imagine these objects. Thus, this may have affected the discrepancies between the two criteria. Therefore, we must investigate an instruction that focuses on the aspects of “imagery” and “imagination.”

Arguably, the self-identification scale does not vary linearly between numbers, while using the VVIQ and self-identification criteria to separate groups may be considered inappropriate. Thus, to confirm that these criteria comprised linear relationships, we performed a correlation analysis using the Pearson correlation (parametric) and Spearman correlation (non-parametric) between the scores for the VVIQ (scores 16–80) and self-identification (scores 1–3). The results revealed that the correlation coefficients were similar for both criteria (Pearson correlation: *r* = 0.251, *p* < 0.01; Spearman correlation: *r_s_* = 0.238, *p* < 0.01). Thus, we assume that the data’s linearity minimally affects the results’ interpretation.

Considering the definition of aphantasia, some possible accounts have been reported—as discussed by Cavedon-Taylor (2022)—including people with aphantasia who lack voluntary imagery (Zeman et al., 2015), lack both voluntary and involuntary imagery (Keogh and Pearson, 2018), and lack conscious imagery but have unconscious imagery (Nanay, 2021). Regarding whether the imagery reduction or absence is only voluntary imagery (Zeman et al., 2015) or both voluntary and involuntary imagery (Keogh and Pearson, 2018), the present results could not examine involuntary imagery because we used the VVIQ or self-identification, which only measure voluntary imagery. The Spontaneous Use of Imagery Scale (SUIS: Reisberg et al., 2003) measures involuntary imagery (Floridou et al., 2022). Keogh and Pearson (2018) used the SUIS to examine people with aphantasia and reported that they exhibited lower SUIS scores. This perspective holds that people with aphantasia may lack both voluntary and involuntary imagery. However, as Cavedon-Taylor (2022) and Floridou et al. (2022) indicated, because SUIS items include both voluntary and involuntary imagery, carefully considering possible definitions of aphantasia to determine whether people with aphantasia lack both voluntary and involuntary imagery is necessary. As the SUIS includes items related to face imagery, such as “When I think about visiting a relative, I almost always have a clear mental picture of them,” considering the definition of aphantasia using the SUIS in terms of consideration of face imagery is also important.

Moreover, based on a study by Jacobs et al. (2018) and Nanay (2021) indicated that people with aphantasia lack conscious imagery but have unconscious imagery. In Jacobs et al.’s (2018) study, participants’ task was to perceive or imagine a geometric shape, such as a triangle or diamond, and then judge whether the random dots presented on the display were within or without the boundaries of the geometric shape. The results revealed that people with aphantasia completed the task as well as the control group. The control group completed the task using conscious imagery, whereas the aphantasia group completed the task using unconscious imagery. Thus, Nanay (2021) suggested that people with aphantasia may lack conscious imagery but may retain unconscious imagery. However, referring to Keogh and Pearson’s (2018) results, Nanay (2021) indicated that whether people with aphantasia retain unconscious imagery is debatable. In Keogh and Pearson’s (2018) study, after participants formed the imagery of red horizontal or green vertical lines (priming stimuli), they were presented with a binocular rivalry display and asked to judge the color that they saw. If the imagery of the priming stimuli was well formed, red horizontal or green vertical lines would appear stronger because they would be the priming stimulus. However, the priming effect did not occur in people with aphantasia. If Jacobs et al.’s (2018) task could be completed using unconscious imagery, it might also have resulted in a priming effect in Keogh and Pearson’s task (Nanay, 2021). However, as the priming effect was not observed in people with aphantasia, it is possible that people with aphantasia do not retain unconscious imagery—as discussed in detail by Blomkvist (2023).

Furthermore, Blomkvist (2023) theorized another account of the cognitive architecture of memory and imagination (she proposed it as the constructive episodic stimulation hypothesis+ [CESH+]; “+” means that she added some processes to the CESH). In this model, various cognitive processes are functionally separated; she proposed indexes for episodic, spatial, and semantic memory. The output of episodic memory is forwarded to the retrieval processes of the visual, auditory, olfactory, and other processes; the output of spatial memory is forwarded to the spatial semantic and spatial episodic retrieval processes; and the output of semantic memory is forwarded to the semantic retrieval process. Further, all these processes are forwarded to the (re)combination process and then output. Seemingly, the CESH+ explains voluntary and involuntary imagery in aphantasia and the subtypes of aphantasia regarding sensory modalities, as discussed in detail by Blomkvist (2023), who indicates that a participant’s intention to trigger control is required to retrieve processes from storage. Moreover, she highlighted that the inability to form voluntary imagery implies that top-down control fails to trigger a relevant retrieval process. In this case, nothing is retrieved and nothing has been forwarded to the (re)combination process. She assumes that the inactivation of these retrieval processes is caused by problems with the memory index, retrieval process, or recombination process. According to her, one possibility is that if there is no problem with the memory index, which can be investigated using brain imaging (e.g., functional Magnetic Resonance Imaging), a reduction in or absence of imagery in aphantasia can be assumed to be attributable to dysfunction at the retrieval stage. She further elucidated the need to consider bottom-up and top-down systems when considering the involuntary imagery of aphantasia. She indicates that people with aphantasia with a reduction in or absence of voluntary imagery are impaired with respect to top-down activation; by contrast, people with aphantasia with a reduction in or absence of voluntary and involuntary imagery are impaired with top-down and bottom-up activation (Blomkvist, 2023). Although possibilities exist for further investigation regarding this point, she posits that the top-down and bottom-up systems are not functioning (the episodic system is functioning) or that the episodic system itself is not functioning. Additionally, because a retrieval process is assumed for each sensory modality in the CESH+, the subtypes of aphantasia may be explained through multisensory imagery.

Mental imagery works specifically for each sensory modality (Belardinelli et al., 2009); we propose that it would be useful to conduct experiments on sensory modalities related to each type (i.e., not only visual imagery tasks but also auditory and tactile imagery tasks, among others). This does not deny the important findings that have been accumulated in the context of visual imagery tasks with aphantasia. To investigate the presence of visual or multisensory aphantasia, reduced visual imagery can be assumed. Therefore, experiments that analyze specific types of visual imagery are required. Furthermore, considering multisensory aphantasia (dysikonesia), experiments on other modalities such as auditory imagery tasks, are also important. That is, if we perform visual imagery tasks, we can observe group differences in task performance between the aphantasia and control groups, irrespective of whether the aphantasia group presents visual or multisensory types of aphantasia. By contrast, if they exhibit only a reduction in visual imagery, their performance on the auditory imagery task may not present differences (or may show small differences) from that of the control group. This may be examined, for example, in following the same/different task (Wu et al., 2006): We assume a visual imagery task wherein, after an animal name is presented in words as Target 1 (T1), an animal picture is presented as T2. Participants create visual imagery (an animal image) from T1 and decide whether T1 (visual imagery created from the animal’s name) and T2 (the animal picture) are the same. Moreover, we assume an auditory imagery task wherein after an animal picture was presented as T1, an animal sound is presented as T2. Participants create auditory imagery (animal sounds) from T1 and decide whether T1 (auditory imagery created from the animal picture) and T2 (animal sounds) are the same (Wu et al., 2006). In a visual imagery task, the performance (reaction time or accuracy) of the visual and multisensory aphantasia groups may be slower or lower than that of the control group, but no group difference between the visual and multisensory aphantasia groups may be observed. By contrast, in an auditory task, no difference in performance (reaction time or accuracy) may exist between the visual aphantasia and control groups. However, the performance of the multisensory aphantasia group may be lower than that of the visual aphantasia and control groups. Thus, the existence of multiple types of aphantasia can be investigated using imagery tasks related to multiple sensory modalities. These findings are crucial, as they indicate the existence of diverse of imagery.

## 5. Conclusion

This study provides data that contribute to characterizing people with aphantasia in terms of visual imagery (i.e., using VVIQ or the self-identification of a reduction in visual imagery), multisensory imagery, cognitive style, and face recognition ability. We found multiple types of visual aphantasia using self-identification of a reduction in visual imagery and face recognition ability, along with the VVIQ. Moreover, multisensory aphantasia and visual aphantasia could be classified using multisensory imagery (QMI). In terms of the verbalizer and visualizer types (VVQ), people with aphantasia exhibited non-specific characteristics pertaining to cognitive style. Although the VVIQ is appropriate for diagnosing visual aphantasia, the use of self-identification and face recognition items reveals the diversity of visual aphantasia. Furthermore, by examining multisensory imagery, we investigated the existence of both multisensory and visual aphantasia. Therefore, these findings contribute to the evidence underscoring the diversity of imagery in people with aphantasia.

## Data availability statement

The datasets presented in this study can be found in online repositories. The names of the repository/repositories and accession number(s) can be found at: https://drive.google.com/drive/folders/1QKunQ2-Z0SAkee6mVirBayh7KNlhx3qC?usp=sharing.

## Ethics statement

The studies involving human participants were reviewed and approved by the Ethics Committee of Fukushima University (approval number: 2021-01). The patients/participants provided their written informed consent to participate in this study.

## Author contributions

JT built the study design, collected the data mainly, and performed analysis. GS and KO also collected the data. DY, SSu, and SSa checked the questionnaires and study protocol. JT drafted the first version of the manuscript. SSu and TH checked the manuscript. TH provided critical revisions. JG provided advice for the manuscript and present survey. All authors developed the study concept and reviewed the manuscript.

## Funding

This work was supported by the Casio Science Promotion Foundation (grant number: 39-50), the Inamori Research Grants to JT, the Japan Society for the Promotion of Science (JSPS) Grant-in-Aid for Scientific Research (22H03910), the Cooperative Research Project of the Research Institute of Electrical Communication, Tohoku University (R03/B11), and the Competitive Research Funds for Fukushima University Faculty (21RI001).

## Conflict of interest

TH was employed by Nippon Telegraph and Telephone Corporation.

The remaining authors declare that the research was conducted in the absence of any commercial or financial relationships that could be construed as a potential conflict of interest.

## Publisher’s note

All claims expressed in this article are solely those of the authors and do not necessarily represent those of their affiliated organizations, or those of the publisher, the editors and the reviewers. Any product that may be evaluated in this article, or claim that may be made by its manufacturer, is not guaranteed or endorsed by the publisher.
