# Supplementary material for: Diversity of aphantasia revealed by multiple assessments of visual imagery, multisensory imagery, and cognitive style
Source: Front Psychol. 2023 Jul 18;14:1174873. doi: 10.3389/fpsyg.2023.1174873 (PMC10403065; doi:10.3389/fpsyg.2023.1174873)
Supplement: Supplementary file 1 [file Data_Sheet_1.docx]

Supplementary Material

# Supplementary Tables

Supplementary Table A1. Results of multiple comparison analysis (QMI) in the aphantasia group.

|  |  | *t* value |  | *p* |  | *Cohen's d* |  |
| --- | --- | --- | --- | --- | --- | --- | --- |
| visual |  |  |  |  |  |  |  |
| clusters 1 vs. 2 |  | *t*(101) = -5.50 |  | .001 |  | -1.95 |  |
| clusters 1 vs. 3 |  | *t*(101) = -9.42 |  | .001 |  | -3.54 |  |
| clusters 1 vs. 4 |  | *t*(101) = -4.71 |  | .001 |  | -2.09 |  |
| clusters 2 vs. 3 |  | *t*(101) = -7.06 |  | .001 |  | -1.58 |  |
| clusters 2 vs. 4 |  | *t*(101) = -0.42 |  | *n.s.* |  | -.83 |  |
| clusters 3 vs. 4 |  | *t*(101) = 4.15 |  | .001 |  | 1.45 |  |
| auditory |  |  |  |  |  |  |  |
| clusters 1 vs. 2 |  | *t*(101) = -8.12 |  | .001 |  | -2.88 |  |
| clusters 1 vs. 3 |  | *t*(101) = -9.87 |  | .001 |  | -3.70 |  |
| clusters 1 vs. 4 |  | *t*(101) = -9.29 |  | .001 |  | -4.13 |  |
| clusters 2 vs. 3 |  | *t*(101) = -3.66 |  | .002 |  | -.82 |  |
| clusters 2 vs. 4 |  | *t*(101) = -3.81 |  | .001 |  | -1.24 |  |
| clusters 3 vs. 4 |  | *t*(101) = -1.21 |  | *n.s.* |  | -.42 |  |
| cutaneous |  |  |  |  |  |  |  |
| clusters 1 vs. 2 |  | *t*(101) = -4.91 |  | .001 |  | -1.74 |  |
| clusters 1 vs. 3 |  | *t*(101) = -8.85 |  | .001 |  | -3.32 |  |
| clusters 1 vs. 4 |  | *t*(101) = -9.96 |  | .001 |  | -4.42 |  |
| clusters 2 vs. 3 |  | *t*(101) = -7.04 |  | .001 |  | -1.58 |  |
| clusters 2 vs. 4 |  | *t*(101) = -8.21 |  | .001 |  | -2.68 |  |
| clusters 3 vs. 4 |  | *t*(101) = -3.16 |  | .001 |  | -1.10 |  |
| kinesthetic |  |  |  |  |  |  |  |
| clusters 1 vs. 2 |  | *t*(101) = -7.15 |  | .001 |  | -2.15 |  |
| clusters 1 vs. 3 |  | *t*(101) = -8.49 |  | .001 |  | -3.19 |  |
| clusters 1 vs. 4 |  | *t*(101) = -10.25 |  | .001 |  | -4.55 |  |
| clusters 2 vs. 3 |  | *t*(101) = -2.90 |  | .027 |  | -.65 |  |
| clusters 2 vs. 4 |  | *t*(101) = -6.17 |  | .001 |  | -2.01 |  |
| clusters 3 vs. 4 |  | *t*(101) = -3.91 |  | .001 |  | -1.36 |  |
| gustatory |  |  |  |  |  |  |  |
| clusters 1 vs. 2 |  | *t*(101) = -6.36 |  | .001 |  | -2.26 |  |
| clusters 1 vs. 3 |  | *t*(101) = -9.78 |  | .001 |  | -3.67 |  |
| clusters 1 vs. 4 |  | *t*(101) = -12.39 |  | .001 |  | -5.50 |  |
| clusters 2 vs. 3 |  | *t*(101) = -6.30 |  | .001 |  | -1.41 |  |
| clusters 2 vs. 4 |  | *t*(101) = -9.93 |  | .001 |  | -3.24 |  |
| clusters 3 vs. 4 |  | *t*(101) = -5.25 |  | .001 |  | -1.83 |  |
| olfactory |  |  |  |  |  |  |  |
| clusters 1 vs. 2 |  | *t*(101) = -2.33 |  | *n.s.* |  | -.83 |  |
| clusters 1 vs. 3 |  | *t*(101) = -5.92 |  | .001 |  | -2.22 |  |
| clusters 1 vs. 4 |  | *t*(101) = -7.74 |  | .001 |  | -3.44 |  |
| clusters 2 vs. 3 |  | *t*(101) = -6.23 |  | .001 |  | -1.40 |  |
| clusters 2 vs. 4 |  | *t*(101) = -7.99 |  | .001 |  | -2.61 |  |
| clusters 3 vs. 4 |  | *t*(101) = -3.48 |  | .004 |  | -1.21 |  |
| organic |  |  |  |  |  |  |  |
| clusters 1 vs. 2 |  | *t*(101) = -2.22 |  | *n.s.* |  | -.79 |  |
| clusters 1 vs. 3 |  | *t*(101) = -4.08 |  | .001 |  | -1.53 |  |
| clusters 1 vs. 4 |  | *t*(101) = -5.86 |  | .001 |  | -2.60 |  |
| clusters 2 vs. 3 |  | *t*(101) = -3.32 |  | .008 |  | -.74 |  |
| clusters 2 vs. 4 |  | *t*(101) = -5.56 |  | .001 |  | -1.81 |  |
| clusters 3 vs. 4 |  | *t*(101) = -3.07 |  | .016 |  | -1.07 |  |

*Note*. Clusters 1 (*n* = 9), 2 (*n* = 55), 3 (*n* = 30), and 4 (*n* = 11) were extracted by cluster analysis in the aphantasia group (*n* = 105).

Supplementary Table A2. Results of multiple comparison analysis (QMI) in the control group with self-identification.

|  |  | *t* value |  | *P* |  | *Cohen's d* |  |
| --- | --- | --- | --- | --- | --- | --- | --- |
| visual |  |  |  |  |  |  |  |
| clusters 1 vs. 2 |  | *t*(298) = -2.00 |  | *n.s.* |  | -.28 |  |
| clusters 1 vs. 3 |  | *t*(298) = -5.10 |  | .001 |  | -.81 |  |
| clusters 2 vs. 3 |  | *t*(298) = -3.80 |  | .001 |  | -.52 |  |
| auditory |  |  |  |  |  |  |  |
| clusters 1 vs. 2 |  | *t*(298) = -7.40 |  | .001 |  | -1.05 |  |
| clusters 1 vs. 3 |  | *t*(298) = -14.37 |  | .001 |  | -2.27 |  |
| clusters 2 vs. 3 |  | *t*(298) = -8.90 |  | .001 |  | -1.23 |  |
| cutaneous |  |  |  |  |  |  |  |
| clusters 1 vs. 2 |  | *t*(298) = -8.96 |  | .001 |  | -1.22 |  |
| clusters 1 vs. 3 |  | *t*(298) = -17.48 |  | .001 |  | -2.77 |  |
| clusters 2 vs. 3 |  | *t*(298) = -11.25 |  | .001 |  | -1.55 |  |
| kinesthetic |  |  |  |  |  |  |  |
| clusters 1 vs. 2 |  | *t*(298) = -9.37 |  | .001 |  | -1.33 |  |
| clusters 1 vs. 3 |  | *t*(298) = -18.15 |  | .001 |  | -2.87 |  |
| clusters 2 vs. 3 |  | *t*(298) = -11.22 |  | .001 |  | -1.54 |  |
| gustatory |  |  |  |  |  |  |  |
| clusters 1 vs. 2 |  | *t*(298) = -13.89 |  | .001 |  | -1.97 |  |
| clusters 1 vs. 3 |  | *t*(298) = -21.06 |  | .001 |  | -3.33 |  |
| clusters 2 vs. 3 |  | *t*(298) = -9.91 |  | .001 |  | -1.37 |  |
| olfactory |  |  |  |  |  |  |  |
| clusters 1 vs. 2 |  | *t*(298) = -9.91 |  | .001 |  | -1.40 |  |
| clusters 1 vs. 3 |  | *t*(298) = -18.34 |  | .001 |  | -2.90 |  |
| clusters 2 vs. 3 |  | *t*(298) = -10.88 |  | .001 |  | -1.50 |  |
| organic |  |  |  |  |  |  |  |
| clusters 1 vs. 2 |  | *t*(298) = -7.84 |  | .001 |  | -1.11 |  |
| clusters 1 vs. 3 |  | *t*(298) = -14.03 |  | .001 |  | -2.22 |  |
| clusters 2 vs. 3 |  | *t*(298) = -8.05 |  | .001 |  | -1.11 |  |

*Note*. Clusters 1 (*n* = 76), 2 (*n* = 142), and 3 (*n* = 83) were extracted by cluster analysis in the control group with self-identification (*n* = 301).

Supplementary Table A3. Results of multiple comparison analysis (QMI) in the control group without self-identification.

|  |  | *t* value |  | *p* |  | *Cohen's d* |  |
| --- | --- | --- | --- | --- | --- | --- | --- |
| Visual |  |  |  |  |  |  |  |
| clusters 1 vs. 2 |  | *t*(2,462) = -13.53 |  | .001 |  | -.89 |  |
| clusters 1 vs. 3 |  | *t*(2,462) = -27.39 |  | .001 |  | -1.80 |  |
| clusters 2 vs. 3 |  | *t*(2,462) = -21.31 |  | .001 |  | -0.91 |  |
| auditory |  |  |  |  |  |  |  |
| clusters 1 vs. 2 |  | *t*(2,462) = -15.01 |  | .001 |  | -.99 |  |
| clusters 1 vs. 3 |  | *t*(2,462) = -34.51 |  | .001 |  | -2.27 |  |
| clusters 2 vs. 3 |  | *t*(2,462) = -29.99 |  | .001 |  | -1.29 |  |
| cutaneous |  |  |  |  |  |  |  |
| clusters 1 vs. 2 |  | *t*(2,462) = -19.34 |  | .001 |  | -1.27 |  |
| clusters 1 vs. 3 |  | *t*(2,462) = -42.57 |  | .001 |  | -2.80 |  |
| clusters 2 vs. 3 |  | *t*(2,462) = -35.70 |  | .001 |  | -1.53 |  |
| kinesthetic |  |  |  |  |  |  |  |
| clusters 1 vs. 2 |  | *t*(2,462) = -23.18 |  | .001 |  | -1.52 |  |
| clusters 1 vs. 3 |  | *t*(2,462) = -43.27 |  | .001 |  | -2.85 |  |
| clusters 2 vs. 3 |  | *t*(2,462) = -30.92 |  | .001 |  | -1.33 |  |
| gustatory |  |  |  |  |  |  |  |
| clusters 1 vs. 2 |  | *t*(2,462) = -23.79 |  | .001 |  | -1.56 |  |
| clusters 1 vs. 3 |  | *t*(2,462) = -47.48 |  | .001 |  | -3.12 |  |
| clusters 2 vs. 3 |  | *t*(2,462) = -36.44 |  | .001 |  | -1.56 |  |
| olfactory |  |  |  |  |  |  |  |
| clusters 1 vs. 2 |  | *t*(2,462) = -20.00 |  | .001 |  | -1.31 |  |
| clusters 1 vs. 3 |  | *t*(2,462) = -43.29 |  | .001 |  | -2.85 |  |
| clusters 2 vs. 3 |  | *t*(2,462) = -35.81 |  | .001 |  | -1.54 |  |
| organic |  |  |  |  |  |  |  |
| clusters 1 vs. 2 |  | *t*(2,462) = -16.32 |  | .001 |  | -1.07 |  |
| clusters 1 vs. 3 |  | *t*(2,462) = -32.99 |  | .001 |  | -2.17 |  |
| clusters 2 vs. 3 |  | *t*(2,462) = -25.65 |  | .001 |  | -1.10 |  |

*Note*. Clusters 1 (*n* = 294), 2 (*n* = 1,100), and 3 (*n* = 1071) were extracted by cluster analysis in the control group without self-identification (*n* = 2,465).

Supplementary Table B1. Results of multiple comparison analysis (VVQ) in the control group without self-identification.

|  |  | *t* value |  | *p* |  | *Cohen's d* |  |
| --- | --- | --- | --- | --- | --- | --- | --- |
| VVQ-visualization |  |  |  |  |  |  |  |
| clusters 1 vs. 2 |  | *t*(2,461) = -6.01 |  | .001 |  | -.32 |  |
| clusters 1 vs. 3 |  | *t*(2,461) = 50.67 |  | .001 |  | 2.81 |  |
| clusters 1 vs. 4 |  | *t*(2,461) = 48.32 |  | .001 |  | 2.90 |  |
| clusters 2 vs. 3 |  | *t*(2,461) = 56.41 |  | .001 |  | 3.13 |  |
| clusters 2 vs. 4 |  | *t*(2,461) = 53.63 |  | .001 |  | 3.22 |  |
| clusters 3 vs. 4 |  | *t*(2,461) = 1.45 |  | *n.s.* |  | .09 |  |
| VVQ-verbalization |  |  |  |  |  |  |  |
| clusters 1 vs. 2 |  | *t*(2,461) = 56.65 |  | .001 |  | 3.02 |  |
| clusters 1 vs. 3 |  | *t*(2,461) = 76.58 |  | .001 |  | 4.25 |  |
| clusters 1 vs. 4 |  | *t*(2,461) = 21.69 |  | .001 |  | 1.30 |  |
| clusters 2 vs. 3 |  | *t*(2,461) = 22.14 |  | .001 |  | 1.23 |  |
| clusters 2 vs. 4 |  | *t*(2,461) = -28.59 |  | .001 |  | -1.72 |  |
| clusters 3 vs. 4 |  | *t*(2,461) = -47.52 |  | .001 |  | -2.95 |  |

*Note*. Clusters 1 (*n* = 704), 2 (*n* = 702), 3 (*n* = 602) and 4 (*n* = 457) were extracted by cluster analysis in the control group without self-identification (*n* = 2,465).

Supplementary Table C1. Correlations among questionnaires in the aphantasia group.

|  | VVIQ | | | | | | | | | | QMI | | | | | | | | | | | | | | | | VVQ | |
| --- | --- | --- | --- | --- | --- | --- | --- | --- | --- | --- | --- | --- | --- | --- | --- | --- | --- | --- | --- | --- | --- | --- | --- | --- | --- | --- | --- | --- |
|  | Full |  | F1 |  | F2 |  | F3 |  | F4 |  | Full |  | Vis. |  | Aud. |  | Cut. |  | Kin. |  | Gus. |  | Olf. |  | Org. |  | Verb. |  |
| VVIQ |  |  |  |  |  |  |  |  |  |  |  |  |  |  |  |  |  |  |  |  |  |  |  |  |  |  |  |  |
| Full |  |  |  |  |  |  |  |  |  |  |  |  |  |  |  |  |  |  |  |  |  |  |  |  |  |  |  |  |
| Factor 1 | .492 | ^**^ |  |  |  |  |  |  |  |  |  |  |  |  |  |  |  |  |  |  |  |  |  |  |  |  |  |  |
| Factor 2 | .477 | ^**^ | -.198 | ^*^ |  |  |  |  |  |  |  |  |  |  |  |  |  |  |  |  |  |  |  |  |  |  |  |  |
| Factor 3 | .531 | ^**^ | -.020 |  | .042 |  |  |  |  |  |  |  |  |  |  |  |  |  |  |  |  |  |  |  |  |  |  |  |
| Factor 4 | .498 | ^**^ | .044 |  | .029 |  | .153 |  |  |  |  |  |  |  |  |  |  |  |  |  |  |  |  |  |  |  |  |  |
| QMI |  |  |  |  |  |  |  |  |  |  |  |  |  |  |  |  |  |  |  |  |  |  |  |  |  |  |  |  |
| Full | .542 | ^**^ | .356 | ^**^ | .267 | ^**^ | .180 |  | .249 | ^*^ |  |  |  |  |  |  |  |  |  |  |  |  |  |  |  |  |  |  |
| Visual | .334 | ^**^ | .503 | ^**^ | .070 |  | -.085 |  | .087 |  | .556 | ^**^ |  |  |  |  |  |  |  |  |  |  |  |  |  |  |  |  |
| Auditory | .318 | ^**^ | .258 | ^**^ | .212 | ^**^ | .016 |  | .133 |  | .741 | ^**^ | .436 | ^**^ |  |  |  |  |  |  |  |  |  |  |  |  |  |  |
| Cutaneous | .466 | ^**^ | .279 | ^**^ | .202 | ^*^ | .203 | ^*^ | .239 | ^*^ | .871 | ^**^ | .417 | ^**^ | .658 | ^**^ |  |  |  |  |  |  |  |  |  |  |  |  |
| Kinesthetic | .487 | ^**^ | .280 | ^**^ | .266 | ^**^ | .176 |  | .228 | ^*^ | .862 | ^**^ | .382 | ^**^ | .633 | ^**^ | .727 | ^**^ |  |  |  |  |  |  |  |  |  |  |
| Gustatory | .383 | ^**^ | .209 | ^*^ | .243 | ^**^ | .130 |  | .161 |  | .850 | ^**^ | .320 | ^**^ | .537 | ^**^ | .714 | ^**^ | .733 | ^**^ |  |  |  |  |  |  |  |  |
| Olfactory | .465 | ^**^ | .222 | ^*^ | .176 |  | .243 | ^*^ | .313 | ^**^ | .768 | ^**^ | .311 | ^**^ | .378 | ^**^ | .675 | ^**^ | .579 | ^**^ | .656 | ^**^ |  |  |  |  |  |  |
| Organic | .451 | ^**^ | .206 | ^*^ | .233 | ^**^ | .275 | ^**^ | .179 |  | .688 | ^**^ | .235 | ^*^ | .334 | ^**^ | .459 | ^**^ | .522 | ^**^ | .517 | ^**^ | .553 | ^**^ |  |  |  |  |
| VVQ |  |  |  |  |  |  |  |  |  |  |  |  |  |  |  |  |  |  |  |  |  |  |  |  |  |  |  |  |
| Verbalization | .085 |  | .042 |  | .022 |  | .118 |  | -.018 |  | .071 |  | .125 |  | .219 | ^*^ | .047 |  | -.014 |  | .035 |  | .054 |  | -.051 |  |  |  |
| Visualization | .264 | ^**^ | .111 |  | .161 |  | .249 | ^*^ | -.021 |  | .248 | ^*^ | .162 |  | .156 |  | .213 | ^*^ | .213 | ^*^ | .166 |  | .153 |  | .255 | ^**^ | .511 | ^**^ |

*Note*. ** *p* < .01, * *p* < .05

Supplementary Table C2. Correlations among questionnaires in the control group with self-identification.

|  | VVIQ | | | | | | | | | | QMI | | | | | | | | | | | | | | | | VVQ | |
| --- | --- | --- | --- | --- | --- | --- | --- | --- | --- | --- | --- | --- | --- | --- | --- | --- | --- | --- | --- | --- | --- | --- | --- | --- | --- | --- | --- | --- |
|  | Full |  | F1 |  | F2 |  | F3 |  | F4 |  | Full |  | Vis. |  | Aud. |  | Cut. |  | Kin. |  | Gus. |  | Olf. |  | Org. |  | Verb. |  |
| VVIQ |  |  |  |  |  |  |  |  |  |  |  |  |  |  |  |  |  |  |  |  |  |  |  |  |  |  |  |  |
| Full |  |  |  |  |  |  |  |  |  |  |  |  |  |  |  |  |  |  |  |  |  |  |  |  |  |  |  |  |
| Factor 1 | .602 | ^**^ |  |  |  |  |  |  |  |  |  |  |  |  |  |  |  |  |  |  |  |  |  |  |  |  |  |  |
| Factor 2 | .699 | ^**^ | .182 | ^**^ |  |  |  |  |  |  |  |  |  |  |  |  |  |  |  |  |  |  |  |  |  |  |  |  |
| Factor 3 | .705 | ^**^ | .277 | ^**^ | .262 | ^**^ |  |  |  |  |  |  |  |  |  |  |  |  |  |  |  |  |  |  |  |  |  |  |
| Factor 4 | .767 | ^**^ | .231 | ^**^ | .513 | ^**^ | .381 | ^**^ |  |  |  |  |  |  |  |  |  |  |  |  |  |  |  |  |  |  |  |  |
| QMI |  |  |  |  |  |  |  |  |  |  |  |  |  |  |  |  |  |  |  |  |  |  |  |  |  |  |  |  |
| Full | .497 | ^**^ | .312 | ^**^ | .535 | ^**^ | .262 | ^**^ | .281 | ^**^ |  |  |  |  |  |  |  |  |  |  |  |  |  |  |  |  |  |  |
| Visual | .477 | ^**^ | .610 | ^**^ | .214 | ^**^ | .292 | ^**^ | .218 | ^**^ | .470 | ^**^ |  |  |  |  |  |  |  |  |  |  |  |  |  |  |  |  |
| Auditory | .286 | ^**^ | .023 |  | .457 | ^**^ | .106 |  | .215 | ^**^ | .755 | ^**^ | .260 | ^**^ |  |  |  |  |  |  |  |  |  |  |  |  |  |  |
| Cutaneous | .365 | ^**^ | .220 | ^**^ | .414 | ^**^ | .188 | ^**^ | .199 | ^**^ | .809 | ^**^ | .306 | ^**^ | .606 | ^**^ |  |  |  |  |  |  |  |  |  |  |  |  |
| Kinesthetic | .349 | ^**^ | .127 | ^*^ | .402 | ^**^ | .215 | ^**^ | .228 | ^**^ | .812 | ^**^ | .282 | ^**^ | .618 | ^**^ | .600 | ^**^ |  |  |  |  |  |  |  |  |  |  |
| Gustatory | .346 | ^**^ | .250 | ^**^ | .408 | ^**^ | .172 | ^**^ | .141 | ^*^ | .839 | ^**^ | .287 | ^**^ | .547 | ^**^ | .640 | ^**^ | .610 | ^**^ |  |  |  |  |  |  |  |  |
| Olfactory | .448 | ^**^ | .248 | ^**^ | .506 | ^**^ | .208 | ^**^ | .290 | ^**^ | .761 | ^**^ | .252 | ^**^ | .455 | ^**^ | .566 | ^**^ | .523 | ^**^ | .629 | ^**^ |  |  |  |  |  |  |
| Organic | .350 | ^**^ | .212 | ^**^ | .368 | ^**^ | .207 | ^**^ | .189 | ^**^ | .751 | ^**^ | .220 | ^**^ | .472 | ^**^ | .494 | ^**^ | .604 | ^**^ | .593 | ^**^ | .466 | ^**^ |  |  |  |  |
| VVQ |  |  |  |  |  |  |  |  |  |  |  |  |  |  |  |  |  |  |  |  |  |  |  |  |  |  |  |  |
| Verbalization | .135 | ^*^ | .009 |  | .122 | ^*^ | .081 |  | .161 | ^**^ | .077 |  | -.007 |  | .092 |  | .025 |  | .062 |  | .022 |  | .147 | ^*^ | .049 |  |  |  |
| Visualization | .096 |  | -.060 |  | .114 | ^*^ | .055 |  | .156 | ^**^ | .173 | ^**^ | .066 |  | .120 | ^*^ | .122 | ^*^ | .122 | ^*^ | .132 | ^*^ | .194 | ^**^ | .135 | ^*^ | .330 | ^**^ |

*Note*. ** *p* < .01, * *p* < .05

Supplementary Table C3. Correlations among questionnaires in the control group without self-identification.

|  | VVIQ | | | | | | | | | | QMI | | | | | | | | | | | | | | | | VVQ | |
| --- | --- | --- | --- | --- | --- | --- | --- | --- | --- | --- | --- | --- | --- | --- | --- | --- | --- | --- | --- | --- | --- | --- | --- | --- | --- | --- | --- | --- |
|  | Full |  | F1 |  | F2 |  | F3 |  | F4 |  | Full |  | Vis. |  | Aud. |  | Cut. |  | Kin. |  | Gus. |  | Olf. |  | Org. |  | Verb. |  |
| VVIQ |  |  |  |  |  |  |  |  |  |  |  |  |  |  |  |  |  |  |  |  |  |  |  |  |  |  |  |  |
| Full |  |  |  |  |  |  |  |  |  |  |  |  |  |  |  |  |  |  |  |  |  |  |  |  |  |  |  |  |
| Factor 1 | .684 | ^**^ |  |  |  |  |  |  |  |  |  |  |  |  |  |  |  |  |  |  |  |  |  |  |  |  |  |  |
| Factor 2 | .759 | ^**^ | .332 | ^**^ |  |  |  |  |  |  |  |  |  |  |  |  |  |  |  |  |  |  |  |  |  |  |  |  |
| Factor 3 | .770 | ^**^ | .371 | ^**^ | .442 | ^**^ |  |  |  |  |  |  |  |  |  |  |  |  |  |  |  |  |  |  |  |  |  |  |
| Factor 4 | .800 | ^**^ | .338 | ^**^ | .549 | ^**^ | .510 | ^**^ |  |  |  |  |  |  |  |  |  |  |  |  |  |  |  |  |  |  |  |  |
| QMI |  |  |  |  |  |  |  |  |  |  |  |  |  |  |  |  |  |  |  |  |  |  |  |  |  |  |  |  |
| Full | .642 | ^**^ | .465 | ^**^ | .581 | ^**^ | .451 | ^**^ | .445 | ^**^ |  |  |  |  |  |  |  |  |  |  |  |  |  |  |  |  |  |  |
| Visual | .566 | ^**^ | .691 | ^**^ | .337 | ^**^ | .343 | ^**^ | .325 | ^**^ | .580 | ^**^ |  |  |  |  |  |  |  |  |  |  |  |  |  |  |  |  |
| Auditory | .470 | ^**^ | .267 | ^**^ | .484 | ^**^ | .324 | ^**^ | .352 | ^**^ | .735 | ^**^ | .353 | ^**^ |  |  |  |  |  |  |  |  |  |  |  |  |  |  |
| Cutaneous | .488 | ^**^ | .303 | ^**^ | .463 | ^**^ | .349 | ^**^ | .364 | ^**^ | .823 | ^**^ | .390 | ^**^ | .601 | ^**^ |  |  |  |  |  |  |  |  |  |  |  |  |
| Kinesthetic | .481 | ^**^ | .295 | ^**^ | .466 | ^**^ | .364 | ^**^ | .334 | ^**^ | .801 | ^**^ | .350 | ^**^ | .565 | ^**^ | .632 | ^**^ |  |  |  |  |  |  |  |  |  |  |
| Gustatory | .461 | ^**^ | .314 | ^**^ | .443 | ^**^ | .334 | ^**^ | .307 | ^**^ | .825 | ^**^ | .357 | ^**^ | .523 | ^**^ | .627 | ^**^ | .630 | ^**^ |  |  |  |  |  |  |  |  |
| Olfactory | .535 | ^**^ | .345 | ^**^ | .480 | ^**^ | .379 | ^**^ | .414 | ^**^ | .797 | ^**^ | .390 | ^**^ | .481 | ^**^ | .613 | ^**^ | .543 | ^**^ | .631 | ^**^ |  |  |  |  |  |  |
| Organic | .413 | ^**^ | .287 | ^**^ | .403 | ^**^ | .300 | ^**^ | .262 | ^**^ | .725 | ^**^ | .298 | ^**^ | .406 | ^**^ | .474 | ^**^ | .518 | ^**^ | .578 | ^**^ | .516 | ^**^ |  |  |  |  |
| VVQ |  |  |  |  |  |  |  |  |  |  |  |  |  |  |  |  |  |  |  |  |  |  |  |  |  |  |  |  |
| Verbalization | .169 | ^**^ | .152 | ^**^ | .091 | ^**^ | .133 | ^**^ | .130 | ^**^ | .088 | ^**^ | .133 | ^**^ | .057 | ^**^ | .084 | ^**^ | .025 | ^**^ | .036 |  | .112 | ^**^ | .024 |  |  |  |
| Visualization | .216 | ^**^ | .178 | ^**^ | .128 | ^**^ | .159 | ^**^ | .182 | ^**^ | .125 | ^**^ | .182 | ^**^ | .084 | ^**^ | .125 | ^**^ | .076 | ^**^ | .051 | ^*^ | .106 | ^**^ | .046 | ^*^ | .278 | ^**^ |

*Note*. ** *p* < .01, * *p* < .05
